# Gene Mutations in Circulating Tumour DNA as a Diagnostic and Prognostic Marker in Head and Neck Cancer—A Systematic Review

**DOI:** 10.3390/biomedicines9111548

**Published:** 2021-10-27

**Authors:** Markéta Hudečková, Vladimír Koucký, Jan Rottenberg, Břetislav Gál

**Affiliations:** 1 Department of Otorhinolaryngology and Head and Neck Surgery, Faculty of Medicine, Masaryk University and St. Anne’s University Hospital, 65691 Brno, Czech Republic; marketa.hudeckova@fnusa.cz (M.H.); jan.rottenberg@fnusa.cz (J.R.); 2 Department of Otorhinolaryngology and Head and Neck Surgery, First Medical Faculty, Motol University Hospital, 15000 Prague, Czech Republic; vladimir.koucky@fnmotol.cz

**Keywords:** head and neck cancer squamous cell carcinoma (HNSCC), circulating tumour DNA (ctDNA), diagnostic biomarker, liquid biopsy

## Abstract

(1) Background: Head and Neck Squamous Cell Carcinoma (HNSCC) is one of the most common malignancies globally. An early diagnosis of this disease is crucial, and the detection of gene mutations in circulating tumour DNA (ctDNA) through a liquid biopsy is a promising non-invasive diagnostic method. This review aims to provide an overview of ctDNA mutations in HNSCC patients and discuss the potential use of this tool in diagnosis and prognosis. (2) Methods: A systematic search for articles published in the English language between January 2000 and April 2021 in the Medline and Scopus databases was conducted. (3) Results: A total of 10 studies published in nine publications were selected and analysed. Altogether, 390 samples were obtained from HNSCC patients, and 79 control samples were evaluated. The most often explored gene mutation in ctDNA was *TP53*. (4) Conclusions: The examination of a larger group of gene mutations and the use of a combination of multiple detection methods contribute to a higher detection rate of mutated ctDNA. More studies are necessary to verify these conclusions and to translate them into clinical practice.

## 1. Introduction

### 1.1. Head and Neck Squamous Cell Carcinoma

Head and neck squamous cell carcinoma (HNSCC) is the sixth most common cancer worldwide, with 890,000 new cases diagnosed annually and 450,000 deaths in 2018 [1]. The risk factors for HNSCC include tobacco exposure, alcohol consumption, betel nut chewing, and infection with oncogenic viruses such as Epstein–Barr virus (EBV) and high-risk human papillomavirus (HPV) strains [2,3]. As much as 80% of oropharyngeal squamous cell carcinoma (OPSCC) cases are attributable to HPV, HPV 16 in particular, depending on the geographic area [4]. Patients with HPV-positive OPSCC generally have a better prognosis compared to HPV-negative patients [5].

The prognosis of HNSCC patients continues to be unfavourable, despite the available surgical and non-surgical therapeutic approaches. The 5-year survival rate of the patients is 25–60% [6]. Early diagnosis is crucial, but in up to 40% of these cases, the disease is diagnosed in advanced stages. The primary treatment modalities are surgery, radiotherapy, chemotherapy, and biological treatment. The choice of treatment strategy is based primarily on clinical and pathological parameters, such as stage of the disease and tumour location.

The long-term follow-up of patients in the post-treatment period requires a thorough clinical examination, frequently accompanied by repeat biopsies and imaging examinations. However, a post-treatment evaluation of the clinical findings is often challenging to interpret due to the postoperative reparative and postradiation changes [7]. These changes are hard to differentiate from the residual or recurrent tumour [7,8]. Unlike other tumours, there is no simple, specific, non-invasive method commonly used in clinical practice to enable effective patient follow-up to monitor the treatment response and detect possible recurrence. Furthermore, there are no reliable methods available for patients with HNSCC to estimate prognosis, treatment response, and enable the tailored oncological treatment beyond TNM classification. This situation endures despite decades of ongoing research of predictive tumour markers and markers for cancer recurrence.

Therefore, much attention has been paid to the genetic background of the tumour transformation. The previously mentioned research activities discovered the most common mechanisms of tumour transformation in the case of HNSCC, but these discoveries are still not applied in everyday practice. The main obstacle, which has to be overcome, is the heterogeneity of the tumour cell population characterised by different genetic profiles. One possible way to detect and utilise the dynamics of the genetic heterogeneity of the tumour cell population is ctDNA analysis via liquid biopsy.

### 1.2. Liquid Biopsy

A liquid biopsy is a non-invasive method, posing a minimal burden on the patients, while providing information on tumour heterogeneity, response to treatment, and residual disease in real time [3,9,10]. This diagnostic tool detects ctDNA, circulating tumour cells (CTC), exosomes and microvesicles [11].

Cell-free DNA (cfDNA) represents short extracellular fragments of DNA. They were first identified by Mandel and Métais, who detected cfDNA in the blood of healthy patients in 1948 [12]. In 1977, increased cfDNA levels were described by Leon et al. in cancer patients [13]. As found out later, however, a high cfDNA level is not cancer-specific. It is also present in other pathological conditions, such as rheumatoid arthritis, systemic lupus erythematosus or liver cirrhosis [14]. Conversely, circulating tumour DNA (ctDNA) concentration correlates with the tumour mass size, vascularity, cellular turnover, location, and treatment response [15,16]. In early-stage cancer and micrometastatic disease, the tumour burden may be very low and, therefore, more likely to yield false-negative ctDNA levels [17].

The ctDNA constitutes a tiny part of cfDNA (<1.0%), which is released from tumour cells into the circulation and carries somatic mutations of primary and secondary tumours [17,18,19]. It is unclear whether ctDNA is released as a by-product of tumour metabolism, or whether it also plays an active role in carcinogenesis, such as releasing fragments of tumour DNA to affect susceptible cells at distant sites [7,20,21]. Due to the rapid metabolisation and elimination of the circulating DNA (by liver, spleen, and kidneys), with a half-life of roughly 10 to 15 min, the ctDNA sheds new light on tumour dynamics in real time [20,22].

Due to its deficient levels, differentiating ctDNA from background cfDNA requires adequately sensitive tests [3]. The identification of somatic mutations in cfDNA was first referred to in the study published by Vasioukhin et al. in 1994 [23]. Since then, the analytical accuracy has considerably improved thanks to advancements in technology. Today, multiple methods are used to detect mutant alleles. Among the conventional techniques are real-time PCR (qPCR), digital PCR-based methods, such as BEAM (bead-based emulsion PCR), ARMS (amplification-refractory mutation system) or and ddPCR (digital droplet PCR) [24,25]. Another option is sequencing, specifically next generation sequencing (NGS) with various modifications, which, thanks to reduced costs and relatively rapid turnaround time, is currently being put under the spotlight [26,27].

There are still many unresolved issues regarding the preanalytical phase of liquid biopsy sample processing, such as the time between blood collection and isolation, centrifugation methods, options of sample freezing and the freezing time, and ctDNA source selection [28,29,30]. In connection with HNSCC and liquid biopsy, cfDNA sources, such as whole blood, plasma or saliva, are discussed in the literature [3,8,27,31,32,33,34,35]. For ctDNA analysis, blood plasma is preferred over serum due to the lower background level of cfDNA, which is 2–24 fold higher in serum [36].

### 1.3. Somatic Mutations of Head and Neck Squamous Cell Carcinoma

According to The Cancer Genome Atlas Network (TCGA), the key somatic mutations in HNSCC are *TP53*, *PIK3CA*, *FAT1* and *CDKN2A* [26,37]. TCGA profiled 279 head and neck squamous cell carcinomas. In HPV-positive HNSCCs, the mutation of the *PIK3CA* oncogene was presented as dominant [37]. HPV-negative tumours exhibited almost exclusively *TP53* mutation and *CDKN2A* inactivation [37].

*TP53* is a tumour suppressor gene and is the most commonly mutated gene in HNSCC—this mutation is found in approximately 50–70% of all tumours [38,39]. In healthy individuals, *TP53* plays a critical role in regulating the cell cycle in response to DNA damage.

Defects of the PI3K-PTEN-AKT pathway, *PIK3CA* specifically, with a prevalence of approximately 6–21% in the whole-exome sequencing of HNSCC, with increased frequency in HPV-positive tumours, is another critical point in the carcinogenesis of the HNSCC. [37,39].

*FAT1* is involved in the regulation of the cell cycle [40,41]. Several recently published whole-exome sequencing studies of HNSCC identified a *FAT1* mutation in 12–23% of patients [37,39].

*CDKN2A*, cyclin-dependent kinase inhibitor 2 A, is a tumour suppressor gene responsible for encoding the protein product p16. P16 plays an essential role in the cell cycle, and any genetic abnormalities inactivating the p16 pathway may confer growth advantages in cells and carcinogenesis [17]. *CDKN2A* mutation was identified in 9–22% of all HNSCC tumours [39,42]. Miracca et al. observed the inactivation of the p16 gene in 7–79% of HNSCC cases studied [43].

## 2. Materials and Methods

### 2.1. Search Strategy

In our review, clinical studies focused on detecting gene mutations in circulating tumour DNA in head and neck squamous cell carcinoma patients were evaluated. Scientific articles published from 2000 to 2021 were systematically screened for in the Medline and Scopus databases by two independent researchers. The search terms included “head and neck tumours”, “head and neck cancer”, “head and neck squamous cell carcinoma”, “head and neck tumours”, “hnscc” or “scchn”; and “ctDNA”, “circulating tumour DNA”, “liquid biopsy”, and “tumour DNA”.

### 2.2. Studies Selection

**Figure 1:**
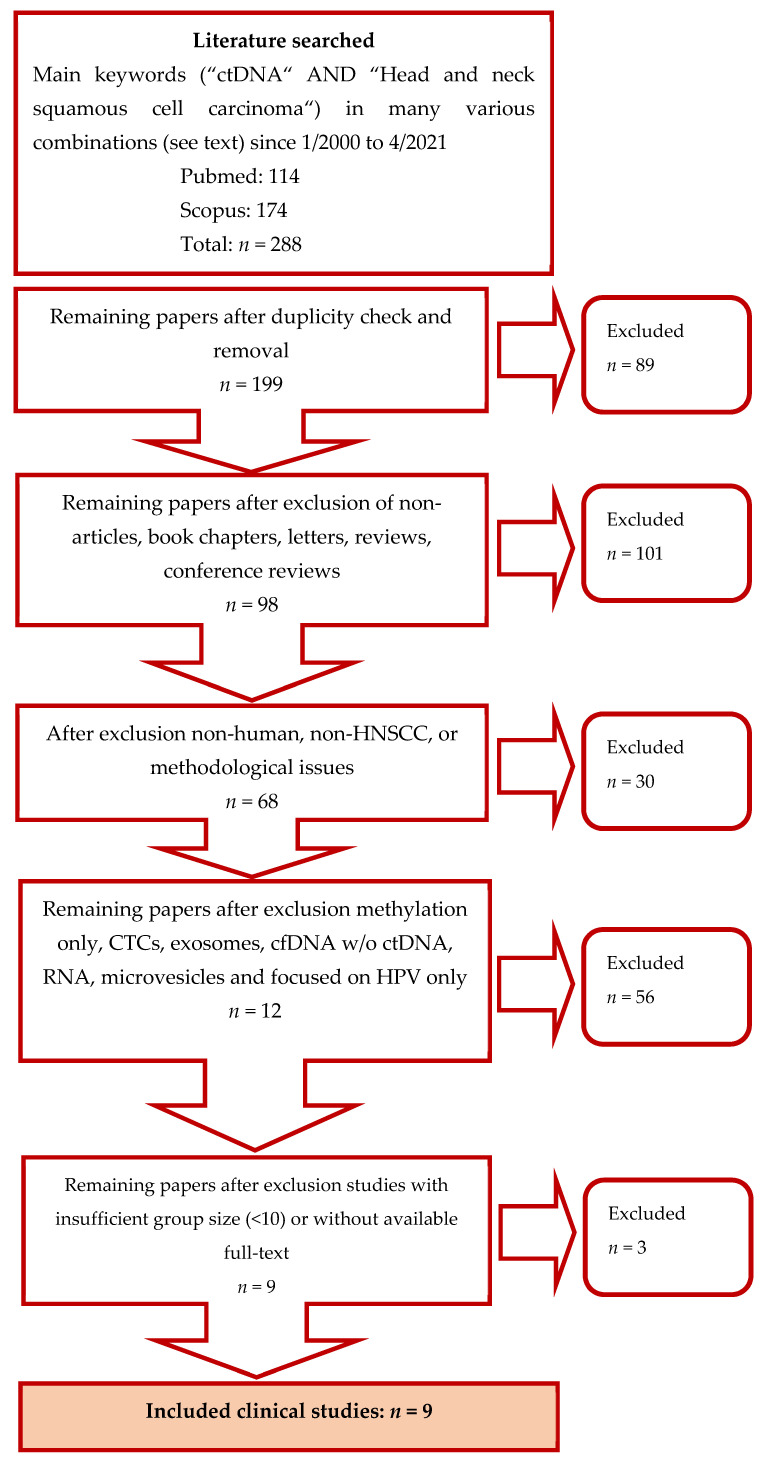
Flowchart illustrating the selection of studies for review.

Only articles published in English were included. Studies not conducted on humans, studies with unclear methods and methodological issues (heterogenic tumour populations including those with tumours other than HNSCC, inconsistent ctDNA collection according to treatment, great variation in time-frames between ctDNA and reference sample collection), studies dealing exclusively with ctDNA methylation, genetic alteration concerning HPV typing only without any further exploration of genome alterations, studies focused only on CTCs, microvesicles, exosomes, RNA, and articles dedicated solely to cfDNA levels without detecting ctDNA mutations were excluded. Detailed illustration of the selection process is shown in Figure 1. The methodological qualities and bias assessment of the final list of studies was performed using the QUADAS-2 tool. QUADAS-2 consists of four categories evaluating study selection, index test, reference standard, and flow and timing by a defined set of questions [44].

## 3. Results

In total, ten studies referred to in nine articles focused on ctDNA mutations in whole blood, plasma, saliva and oral rinses in patients with HNSCC were evaluated after the literature search (Table 1). The largest study cohort comprised 93 patients [31], whereas the smallest consisted of 11 patients only [34]. Three hundred and ninety samples obtained from patients with HNSCC and 79 control non-tumour samples were analysed using various methods. In all of these studies, patients with HNSCC across anatomical locations were included (oral cavity, oropharyngeal, hypopharyngeal or laryngeal SCC). In one study, the primary site was not specified [35], and one group also included the patients with salivary gland and thyroid cancers [27]. This sub-group of patients was, however, not included in specific analyses of ctDNA. According to the TNM staging system, patients across the spectrum of stages, with prevailing advanced cases, participated in the studies. In some of the studies, the tumour stage was not specified [27,33,34,35].

The source of ctDNA was predominantly plasma, namely in 8 out of 10 studies. The most frequently examined gene mutations were *TP53*, *PIK3CA*, *CDKN2A* and *NOTCH1*. HPV positivity was detected in 77 patients, but this information was not provided in several publications. The PCR (PCR, real-time PCR, ddPCR) techniques and sequencing, with a predominance of NGS, were used for analysis. The common main objective of all of the studies was to verify the applicability of ctDNA as a diagnostic and prognostic factor in patients with HNSCC. The specific characteristics of the studies are given in Table 1.

The overview of most frequently mutated genes, regardless of HPV status and the analytical method used, are listed in Table 2. It includes only those studies in which ctDNA was analysed in patients before the commencement of treatment, and studies in which individual somatic mutations were quantified.

## 4. Discussion

The early diagnosis and detection of recurrent HNSCCs are essential for the further clinical course of the disease and treatment. A thorough clinical examination and necessary biopsy under local or general anaesthesia and imaging (usually contrast-enhanced CT imaging, MRI or PET/CT) are used to diagnose patients at present. These examinations may pose a heavy burden for the patients, especially in the phase of clinical follow-up when attempting to detect tumour persistence or recurrence. In this summary, we present studies published in the last 20 years that explore the potential applicability of gene mutations’ detection in DNA obtained from plasma, serum or whole blood, or oral rinses. The objective was to summarise the current knowledge of ctDNA mutations in HNSCC as a diagnostic and prognostic marker.

Although in reviewed studies the primary source of ctDNA was blood (plasma, serum, whole blood), some of the studies also used oral rinses. Wang et al. analysed ctDNA in oral rinses and plasma, and the results were evaluated regarding sites of the primary cancer [31]. Mutations of *FBXW7*, *NRAS*, *HRAS*, *PIK3CA*, *TP53*, *CDKN2A* genes were investigated. As expected in the study, the highest detection of DNA fragments in oral rinse was in the oral cavity SCC, namely 100%. In contrast, in the other primary sites (oropharyngeal, hypopharyngeal and laryngeal SCCs), the detection was 47–70%. The best results were achieved in the combined detection of mutant DNA in plasma and oral rinse, namely 96% on average. The same conclusion was also achieved by Perdomo et al. in the LA study, where only the *TP53* mutation was evaluated [32]. The mutation was detected in 46.2% of oral cavity tumours and in 60% of oropharyngeal cancer in oral rinses, while in plasma, the *TP53* mutation was detected in 8% of samples only (*n* = 3, two tumours were located in the larynx, and one case exhibited the tumour in multiple sites without further specification). These results indicate that the detection of tumour DNA, whether in plasma or oral rinses, correlates with the tumour site. More groups of patients with a determined oral rinse collection would be necessary to confirm this proposition. In the case of the non-specified primary tumour site in the study by Wang et al., however, the detection of ctDNA in plasma is higher than in saliva, namely 87% to 76% [31]. This fact points to plasma as the more promising and universal ctDNA source for detection, particularly in carcinomas of unknown primary.

Various analytical methods were also applied in the study by Mes et al., who combined the detection of somatic mutations, HPV-DNA, and CNA (copy number aberrations) [45]. If the analysis focused exclusively on ctDNA in plasma, the sequencing method detected tumour DNA in 67% of samples. When all three methods were combined, the detection rate increased to 78%. The HPV status, or the tumour site, did not affect detection, but a positive correlation was observed between the TNM stage, and CNAs and somatic mutations in plasma. The analysed groups of HNSCC cancer patients have not been large enough to confirm or disprove this finding. Nevertheless, there is a correlation between the CNA burden and disease stage described by Hieronymus et al. in colorectal cancer [47]. In the group of 40 patients described by Mes et al., the fourth stage of cancer strongly prevailed (I = 2, II= 4, III = 6, IV = 28) [45]. The question is whether such a CNA burden would also be described in a group of patients with earlier stages of the disease.

The potential method for the selection of investigated ctDNA gene mutations appears to be cardinal. According to the studies, the highest yield is achieved in the selection based on gene mutations of primary cancer. The key mutations in tumour tissue were recently described in The Cancer Genome Atlas: *TP53* (72 %), *PIK3CA* (21%), *FAT1* (23%) and *CDKN2A* (22%). Our review comprises both studies focused on one gene only, most commonly *TP53*, and on studies detecting a whole set of genes. The studies we analysed indicate that the number of explored genes correlates with ctDNA detection (Table 2). There is also a correlation between this finding and the conclusions by Schwaederle et al., who looked into specific mutations of ctDNA in various types of cancer, including 25 patients with HNSCC [35]. Based on their observations, 88% of patients with HNSCC had ctDNA mutations, namely in 40% of patients 1–3 mutations were detected, and in 48% of patients more than three mutations were present, which was the highest number of mutations compared to all of the other cancers (gastrointestinal tract, brain, lung, and breast cancers). This fact is attributed to the high heterogeneity of HNSCC compared to other cancers.

Moreover, the tissue biopsy specimen taken from one site fails to describe enormous genetic diversity in the primary tumour over time, or between the primary tumour and metastases, or tumour relapse. This fact also explains why, in the study by Porter et al., 73% of patients with advanced tumours had ctDNA mutations detected in blood, which were not analysed in the examined tumour sample [27].

There are two types of ctDNA analysis. The first one is the analysis of tumour tissue followed by cfDNA examination conducted only if the mutations are detected. In the second case, the study of tumour tissue is skipped, e.g., for its inaccessibility, and cfDNA is analysed straight away; this is, however, a more challenging process requiring high specificity and sensitivity techniques. In three of the reviewed studies, the mutation is first analysed in tumour tissue, and only if detected is it also searched for in the patient’s plasma [31,32,34]. The ctDNA detection, compared to mutation detection in tumour tissue, ranges between 8 and 87%. Apart from numerous preanalytical factors, this discrepancy could also be explained by a finding published by Fiala et al., who calculated that a tumour volume of at least 1 cm^3^ is needed for ctDNA to be detected [48]. This fact is crucial for the applicability of ctDNA as a potential biomarker to diagnose cancer in asymptomatic patients, or to early detect the recurrence of the earlier diagnosed cancer.

Recurrent and metastatic HNSCC was the topic of the study by Galot et al., who in a cohort of 39 patients presented a considerably higher ctDNA detection in patients with metastatic HNSCC (70%) compared to patients with locoregional recurrent HNSCC (30%) [33]. This fact can limit the potential use of ctDNA as a diagnostic tool in patients with locoregional recurrent HNSCC without metastases. Another study focused directly on the use of ctDNA in practice in patients with advanced HNSCC was the study by Porter et al., who explored the possibility of identifying usable mutations in ctDNA to design the targeted therapy [27]. Eight patients received matched targeted therapy (MTT), three of whom had stable disease at the time of the study. In two of the patients, *BRAF* mutation was treated by dabrafenib/trametinib and vemurafenib. In the third patient, *EGFR* mutation was treated by cetuximab. Cetuximab therapy was also investigated by Braig et al., who studied the disease response and acquired *RAS* mutations in ctDNA in patients undergoing chemotherapy combined with cetuximab [46].

Interestingly, they discovered that acquired *RAS* mutation was found in patients demonstrating HNSCC progression. It was, however, not detected in patientswith a stable disease. This fact suggests the potential reasons behind the acquired resistance of HNSCC to therapy by cetuximab.

The use of liquid biopsy and ctDNA in HNSCC has potential in diagnosis and prognosis, whether for primary, metastatic or recurrent disease. It is also worth mentioning that the studies we compared did not describe any correlation between ctDNA mutations and the overall survival of patients with HNSCC, and no association of *TP53* mutation in tumour tissue with age, exposure to smoking, alcohol, and primary tumour site was found either. However, Wilson et al. showed, in a cohort of 75 HNSSC patients, a decreased OS of patients with detected ctDNA alterations. Moreover, alterations in DNA repair genes in ctDNA correlated with a worse OS both in univariate and multivariate analyses. Interestingly, the study found no correlation of DNA repair genes alterations in tumour DNA and patient prognosis [49]. Additionally, the study used a wide variety of time gaps between the assessment of ctDNA and tumour DNA.

Despite the potential to use the liquid biopsy as a possible tool for tumour diagnosis, recurrence identification, or to predict the treatment response, there is much more to discover before this method can be used in everyday clinical practice for HNSCC. First of all, the methodological problems have to be solved, and the procedure of liquid biopsy has to be optimised and standardised in both the pre-analytical and analytical aspects.

Even if the technological problems are solved, the liquid biopsy results may be influenced by patients’ metabolic activity and velocity of circulating DNA decomposition. The deoxyribonuclease activity in human plasma is relatively high, but it was discovered it significantly drops in cases of colorectal cancer [50] and prostatic cancer [51]. This mechanism is still not fully understood and must be clarified before the method can be safely used in clinical practice. The information about ctDNA metabolism may also change our view on the relationship between ctDNA and cancer. Therefore, clinical research has to be focused not solely on the ctDNA levels and their genetic structures, but also on the whole metabolic chain regulating the level of circulating DNA in the blood.

The most practical application of ctDNA detection in HNSCC patients is tertiary prevention and tumour recurrence detection. It has been shown that the level of ctDNA correlates with tumour size, but it is still not clear whether monitoring ctDNA levels would show sufficient sensitivity. The clinical data from the study conducted in patients with recurrent gastric cancer show a high sensitivity of ctDNA detection [52], but the method of the whole genome sequencing (WGS) used in this study is rather costly and slow for routine clinical application. Moreover, the study also showed inconsistent results of ctDNA positivity during the follow-up periods. For example, one patient showed ctDNA positivity early after the operation but later became ctDNA-negative despite the apparent tumour recurrence. This indicates a much more complicated relationship between ctDNA positivity and the size of a tumour in an individual case, which still forms an obstacle to the straightforward use of ctDNA as a marker of tumour recurrence.

The other potential use of ctDNA is to detect the genetic background of tumour transformation in patients with HNSCC. As all tumour cells release fragments of their DNA into the bloodstream, we will presumably have a mixture of genetic material originating from all, or at least significant types, of genetic clones forming the overall tumour mass. This might help to overcome the problem of the genetic heterogeneity of the tumour cell population and, therefore, pave the way to the applicability of therapeutical tailoring based on its genetic background. It is possible to detect particular ctDNA tumour genotypes, but it is not clear whether we could also measure their concentrations. In some pilot studies in patients with follicular lymphoma [53], a quantitative analysis of the tumour subclones based on ctDNA was completed by NGS. The study results were ambiguous as only half of the ctDNA samples correlated with the cell subclone proportion in the actual tumour mass. The study showed that the number of genetic subclones in the tumour mass detectable by NGS varied from 2 to 20. The authors recommended the continuation of the same research on a larger patient population. Even if possible, there remains a big question of whether these concentrations will represent the proportion of individual cell clones in the tumour population in HNSCC. However, the number of genetic subclones might be the independent prognostic factor that may signal potential radioresistance and chemo-refractoriness.

Therefore, this study design is also applicable in the clinical practice of HNSCC cases because approximately half of the cases respond well to chemo- and radiotherapy. Presumably, these cases would have a low number of tumour subclones detectable from which the refractoriness resistance could arise.

## 5. Conclusions and Future Research

According to the available literature, ctDNA is a highly sensitive genetic biomarker that directly reflects tumour burden in melanoma [54], pancreatic [55], colorectal [24], lung [55], breast [56] and prostate [55] cancers. Nevertheless, there are not enough studies for HNSCC with a larger cohort of patients with a clearly defined area of interest, standardised preanalytical and analytical methods, and conditions set for sample collection, processing and storage.

The detection of ctDNA as a biomarker of recurrence in HNSCC during the post-treatment follow-up is likely to have a more suitable application than its use as a diagnostic marker of primary cancer. Future research should consider two main topics. Available studies use different ctDNA analytical methods and these methods provide different results depending on the type and clinical condition of the tumour. Despite the usability of ctDNA analysis in patients with other histological types of cancer, in the case of HNSCC, the methods are not unified yet. To achieve consistent analytical results in patients with HNSCC, further analyses of specificity, sensitivity, and concordance with tumour samples are needed for each analytical method together with a critical evaluation of the pre-analytical sampling phase. Once these data are known, or when the procedures are sufficiently consolidated, another part of clinical research should include prospective studies evaluating the significance of these methods, especially in the tertiary prevention of HNSCC, i.e., the early detection of possible cancer relapse compared to the current methods of clinical monitoring.

## Figures and Tables

**Table 1:** Characteristics of studies using ctDNA as a biomarker.

Study	Ref.	Groups			Tumor		HPV + Patients	DNA Source	Genes						Assay Type	Focus of the Study	Results
		Patients		Controls	Site	STAGE			*TP53*	*PIK3CA*	*NOTCH1*	*CDKN2A*	*CASP8*	Other			
Schmidt et al., 2018	[3]	29		10	OPSCC, OSCC, HPSCC, LSCC	III-IV	14	plasma		x				no	Plex-PCR^TM^	Usability of Plex-PCR^TM^ technology *PIK3CA* mutation detection p.E545K in HNSCC samples	The results of the pilot study support the applicability of using allele-specific technologies for ctDNA testing in HNSCC.
Porter et al., 2019	[27]	60 (48 HNSCC)		x	OPSCC, SG, TG, other HNC	III, IV	15 of OPSCC	blood samples	x	x	x			ARID1A, panel Guardant360 (73-gene ctDNA NGS platform)	NGS	Characterizing a ctDNA blood sample in advanced patients with HNSCC to identify a useful mutation and elucidate a potential role in treatment.	Of the patients with usable mutations, 13% (n = 8) received appropriate targeted therapy (MTT). Result: 3 stable diseases (37.5%), 3 progressive diseases (37.5%) and 2 (25%) were not evaluated at follow-up
Wang et al., 2015	[31]	47		x	OSCC, OPSCC, HPSCC, LSCC	I-II: 20, III-IV: 3	30	plasma, oral rinses	x	x		x		FBXW7, *NRAS*, *HRAS*	PCR	Detect genetically modified DNA in the saliva or plasma of HNSCC patients with tumours of various stages and anatomical sites.	Tumour DNA was found in 76% of saliva (93 pts). Tumour DNA was found in 87% of plasma (47 pts.).
Perdomo et al., 2017	[32]	ARCAGE	36	x	OSCC, OPSCC, LSCC	I: 6, II: 8, III: 8, IV: 14	0	plasma	x		x	x	x	PTEN	sequencing	To provide a comprehensive evaluation of the presence of ctDNA in plasma and oral washes in HNSCC patients in early and late stages. Two strategies for detecting ctDNA mutations.	ARCAGE: Tumour-specific mutations in 5 genes in plasma (42% of cases), most of them (67%) were early-stage cases
		LA	37	49	OSCC, OPSCC, LSCC, over.	III-IV	1	plasma, oral rinses	x					no			LA: mutation concordance in tumour tissue, plasma and mouthwash only in 1 sample; in 4 cases, the conformity of the mutation in the oral lavage and tumour sample
Galot et al., 2020	[33]	39		x	OSCC, OPSCC, HPSCC, LSCC	x	5 of OPSCC	plasma	x	x	x		x	EGFR, ERBB3, AXL, CSF1R, RET, PIK3R1, AKT1, MAPK1, NF1, STK11, AURKA, MYC, NFE2L2, KMT2C, KMT2B, CREBBP, NSD1, SMARCA4, SMARCA2, ARID1A, BRCA1, MLH1, MSH2, CTNNB1, *FAT1*, FAT4, NOTCH2, PTCH1	NGS	Applicability of liquid biopsy for characterization of the mutational environment of recurrent/metastatic HNSCC	Significantly higher probability of ctDNA detection in patients with metastatic disease than patients with locoregional recurrence alone (70% vs 30%).
Coulet et al., 2000	[34]	11		x	x	x	x	plasma	x					no	PCR	Quantification cfDNA, evaluation ctDNA and analysis clinical significance.	Plasma DNA concentration was measurable at 35%. Tumour DNA detected in plasma in 18% (ctDNA)
Schwaederle et al., 2017	[35]	25		x	HNC	x	x	plasma	x	x				EGFR, BRECA2, APC, MET, *BRAF*, ERBB2, MYC, NF1, ARID1A, SMAD4, BRCA2, FGFR2, BRCA1, PDGFRA, ALK, AR, FGFR1	NGS	The study specifically examines mutations in the ctDNA of various cancers. Included 25 patients with HNSCC	88% had a mutation. 40% have 1–3 mutations, 48% have > 3 mutations
Mes et al., 2020	[45]	40		20	OSCC, OPSCC, HSCC, LSCC, other	I = 2, II = 4, III = 6, IV = 28	10 of OPSCC or unknown primary locality	plasma	x	x	x	x	x	AJUBA, *FAT1*, *FBXW7*, *HRAS*, KMT2D, NSD1, PTEN	deep sequencing	Combined detection of somatic mutations, HPV-DNA and CNA using the same sequencing library and recovery assessment in pre-treatment HNSCC patient plasma samples.	The combination of analysis of CNA (copy number aberrations), HPV and somatic mutations in plasma contributes to higher sensitivity than individual modalities.
Braig et al., 2016	[46]	20		x	HNSCC	II: 2, III: 3, IV: 15	2	serum						RAS	NGS	Responses and acquired mutations in the *RAS* ctDNA gene of HNSCC patients treated with cetuximab plus chemotherapy	Patients with the progressive disease showed a *RAS* mutation. Patients without progression did not show any additional *RAS* mutation

One size of the patient group; 2 tumour sites: OSCC—oral squamous cell carcinoma, OPSCC—oropharyngeal squamous cell carcinoma, HPSCC—hypopharyngeal squamous cell carcinoma, LSCC—laryngeal squamous cell carcinoma, SG—salivary glands, TG—thyroid gland, HN—head and neck carcinoma without further specification.

**Table 2:** Overview of detected ctDNA mutations in blood and detection of specific mutations.

Study		Ref.	No of Patients	Overall Percentage Independent on HPV Typing						
				ctDNA	Investigated Gene Mutations	*TP53*	*PIK3CA*	*NOTCH1*	*CDKN2A*	*CASP8*
Schmidt et al., 2018		[3]	29	31%	PIK3CA		31%			
Wang et al., 2015		[31]	47	87%	HPV16/18, *FBXW7*, *NRAS*, *HRAS*, *PIK3CA*, *TP53*, *CDKN2A*	no data available				
Perdomo et al., 2017	ARCAGE	[32]	36	42%	*TP53*, *NOTCH1*, *CDKN2A*, CASP8, PTEN	31%		3%	6%	3%
	LA		37	8%	*TP53*	8%				
Coulet et al., 2000		[34]	11	18%	*TP53*	18%				
Mes et al., 2020		[45]	40	67%	AJUBA, CASP8, *CDKN2A*, *FAT1*, *FBXW7*, *HRAS*, KMT2D, NOTCH1, NSD1, *PIK3CA*, PTEN, *TP53*	no data available				
